# Neuron Names: A Gene- and Property-Based Name Format, With Special Reference to Cortical Neurons

**DOI:** 10.3389/fnana.2019.00025

**Published:** 2019-03-21

**Authors:** Gordon M. Shepherd, Luis Marenco, Michael L. Hines, Michele Migliore, Robert A. McDougal, Nicholas T. Carnevale, Adam J. H. Newton, Monique Surles-Zeigler, Giorgio A. Ascoli

**Affiliations:** ^1^Department of Neuroscience, Yale School of Medicine, New Haven, CT, United States; ^2^Yale Center for Medical Informatics, New Haven, CT, United States; ^3^Institute of Biophysics, National Research Council, Palermo, Italy; ^4^Department of Physiology and Pharmacology, SUNY Downstate Medical Center, Brooklyn, NY, United States; ^5^Bioengineering Department and Center for Neural Informatics, Krasnow Institute for Advanced Study, George Mason University, Fairfax, VA, United States

**Keywords:** neuron classification, terminology, axons, dendrites, brain regions, genomics

## Abstract

Precision in neuron names is increasingly needed. We are entering a new era in which classical anatomical criteria are only the beginning toward defining the identity of a neuron as carried in its name. New criteria include patterns of gene expression, membrane properties of channels and receptors, pharmacology of neurotransmitters and neuropeptides, physiological properties of impulse firing, and state-dependent variations in expression of characteristic genes and proteins. These gene and functional properties are increasingly defining neuron types and subtypes. Clarity will therefore be enhanced by conveying as much as possible the genes and properties in the neuron name. Using a tested format of parent-child relations for the region and subregion for naming a neuron, we show how the format can be extended so that these additional properties can become an explicit part of a neuron’s identity and name, or archived in a linked properties database. Based on the mouse, examples are provided for neurons in several brain regions as proof of principle, with extension to the complexities of neuron names in the cerebral cortex. The format has dual advantages, of ensuring order in archiving the hundreds of neuron types across all brain regions, as well as facilitating investigation of a given neuron type or given gene or property in the context of all its properties. In particular, we show how the format is extensible to the variety of neuron types and subtypes being revealed by RNA-seq and optogenetics. As current research reveals increasingly complex properties, the proposed approach can facilitate a consensus that goes beyond traditional neuron types.

## Introduction

Accurate terminology for neurons is increasingly needed for communication of research on the nervous system and the establishment of databases that can give access to data about neurons and their properties. Here we show how this effort can be enhanced by combining neuron databases based on traditional names with new research on genes and neuron properties, in a format in which multidisciplinary properties are part of the name. This extended format facilitates the investigation of a given neuron type within the context of all its properties, while at the same time providing an orderly listing of neurons within databases of neurons of different brain regions. This is particularly advantageous in support of studies of the complex properties of neurons in different regions of the cerebral cortex.

Most terms for neurons are holdovers from the nineteenth century, when it was discovered that nerve cells appear as distinct types based on the structure and location of their axons, dendrites and cell bodies. The terms varied in an idiosyncratic way among investigators, based on personal impression and imagination. Small cells were often called “granules,” and large cells sometimes given the discoverer’s name, such as “Purkinje cell” or “Betz cell.” All attempts to bring order into this daunting terminological jungle originate in the vast overview contained in Ramón y Cajal’s (1911) great work *Histologie du système nerveux de l’homme & des vertébrés*.

The pace of modern neuroscience research is now carrying us far beyond this strictly anatomical base. In particular it is providing new properties based on gene expression and functional characteristics. We have been developing archives of neurons and their properties, in which the names are listed separately from their properties. However, the properties are increasingly central to the identity of a neuron type. Incorporating these multimodal properties in the neuron name will have distinct advantages, so that a neuron can be readily searched and recognized, to facilitate research into increasingly complex types. The present aim is to outline a framework for achieving this.

### Background

The SenseLab^1^ suite of databases, initiated in 1993 and building on publications on synaptic organization of local regions, contains NeuronDB, which has focused on the terminology and properties of many of the most highly investigated neurons^2^ (Mirsky et al., 1998; Crasto et al., 2007). As stated at the outset (Shepherd et al., 1998, p. 466):

“… NeuronDB … is a tool for enabling the user to understand the significance of a molecular property within the context of other properties contributing to the functions at a particular site within a particular neuron. This is a goal, not only for neuroscientists, but also for molecular biologists studying gene function in the emerging fields of functional genomics and pharmacogenomics.”

With this approach, a neuron name is closely related to its multimodal properties, a principle that will be used in expanding to a systematic, properties-based, terminology outlined in this paper.

An early effort in developing a systematic neuron terminology occurred within a Brain Architecture Knowledge Management System (BAMS) (Bota and Swanson, 2007, 2008, 2010), which has been later related to rat and mouse connectomes (Bota et al., 2012a,b). The NeuronDB approach was greatly expanded by an effort sponsored by the Society for Neuroscience with the Neuroscience Information Framework (NIF) (Gardner et al., 2008), a web-based data and knowledge archive, with a section entitled Neurolex Neuron aimed at providing a list of all the known neurons in vertebrates and invertebrates, including those in BAMS. Accompanying the list were entries for each neuron containing further information on such properties as axon myelination, dendritic branching, soma site, neurotransmitters, etc., A series of articles related to this effort developed an ontological approach to the terminology of the neurons and their properties (Bug et al., 2008; Hamilton et al., 2012; DeFelipe et al., 2013; Larson and Martone, 2013; Polavaram and Ascoli, 2017). Many of these principles have been tested for neuron types from the rodent hippocampal formation in Hippocampome.org (Wheeler et al., 2015; Hamilton et al., 2017), which is closely related to the present account. Detailed coverage of neurons has been available in *The Synaptic Organization of the Brain* (Shepherd, 1974, 2004), and from the perspective of organization of neurons into microcircuits in the *Handbook of Brain Microcircuits* (Shepherd and Grillner, 2018).

The new era based on the methods of single cell transcriptomics and optogenetics is revealing the genes expressed by, and the electrophysiological properties of, morphologically identified neuron types. Here we build on the multimodal representation of neurons in several databases: NeuronDB, which started with representations of morphology, neurotransmitters, neurotransmitter receptors, and ion channels; ModelDB, which contains realistic neuron models reproducing the firing properties; and NeuroMorpho.Org (Ascoli et al., 2007; Akram et al., 2018), which contains reconstructions of the morphology of the cell types.

It is now timely to incorporate the new data on multimodal properties. The aim of the BRAIN Initiative Cell Census Consortium (Ecker et al., 2017) is “developing, validating, and scaling up emerging genomic and anatomical mapping technologies for creating a complete inventory of neuronal cell types and their connections in multiple species and during development.” The present proposal goes beyond genomics and anatomy, with the ultimate aim of the Consortium: a hierarchical organization of multimodal features in neuron names:

“Finally, the importance of establishing a common cell type nomenclature across species cannot be overstated …. The nomenclature could follow a hierarchical order, starting at the highest level: the species, then the brain region annotated based on a unified anatomical reference atlas system with cross-correlations among species, and then the *cell type as defined by a multimodal feature set (including locational, molecular, morphological, physiological, and ontological features)* (ed. italics)…. The nomenclature should be a culmination of knowledge gained about the cellular organization of the nervous system.” (Ecker et al., 2017, p. 551)

A similar initiative is reported in “The NIF ontology: brain parcels, cell types, and methods” (Gillespie et al., 2018). It recognizes the need for multiple techniques to reach a consensus “about even a single aspect of a cell type.” It also aims to provide a knowledge base for neuron types “characterized by accumulated knowledge” regarding multiple phenotypes including “species, anatomical, molecular, morphological, physiological, synaptic and projection targets.”

Finally, a major new initiative by the Allen Brain Institute (see Tasic et al., 2018) uses single cell RNA-seq, stochastic neighbor embedding (SNE) and connectivity methods to establish neuron identities on the combined basis of genes, markers, laminar localization and hodology. As will be shown, the format for reporting these results fits well with the present proposal. The multidisciplinary aims of these initiatives are thus shared with the current databases, underlying the timeliness of combining them in the present proposal.

## Initial Considerations

With regard to the format of the neuron nomenclature, the approach used in NeuronDB and the NIF is to anchor the neuron in the region containing its cell nucleus (for most purposes the cell body). This sets up a parent-child relation for all neurons belonging to this region. In listing neurons in the brain, neuron types are thus all contained in alphabetical order within their appropriate region. This avoids any confusion about the identity of disparate neurons with similar names, such as granule cell, stellate cell, or pyramidal cell.

After the region, the neuron needs to be situated in its subregion, followed by its properties. In general, as indicated above, a consensus is emerging on the main categories of multimodal properties. As outlined in Table 1, the general order begins with defining the anatomical location and neuron morphology, followed by gene and molecular markers, physiological properties, and neurotransmitter. These are all properties of the neuron itself (“intrinsic”).

**Table 1:** Categories of multimodal properties, for example, of a neocortical pyramidal neuron and interneuron in the primary motor area M1.

	Anatomical Properties					Functional Properties			
		
Naming system	Region	Subreg	Layer	Conn	Name	Genes	Peptides	Physiol	Trans
**Pyramidal neurons**									
Traditional	Neocortex	Ml	L2/3		Pyramid				
NIF/NeuronDB	Neocortex	M1	L2/3	IT	Pyramid			{non-adapt	GLU}
Proposed	Neocortex	M1	L2/3	IT	Pyramid			{non-adapt	GLU}
									
**Interneurons**									
Traditional	Neocortex	M1	L2/3		Basket				
NIF/NeuronDB	Neocortex	M1	L2/3	INT	Basket		{SST	burst	GABA}
Proposed	Neocortex	M1	L2/3	INT	Basket	*Pvalb*	{SST	burst	GABA}


*The table compares the formats for the relatively stable (anatomical) properties and functional properties used in the nomenclature in traditional use, NeuronDB/NIF NeuroLex, and the present proposal. Brackets {} indicate that in most current schemes the functional properties are stored in a separate database; in the present proposal they are part of the neuron name itself. Subreg, subregion; Conn, connectivity; Physiol, physiology; Trans, transmitter; L, layer; IT, intratelencephalic; INT, interneuron; non-adapt, non-adapting impulse firing; GLU, glutamate; GABA, gamma-amino butyric acid. References will be added to document critical properties.*

An inconvenience for any nomenclature scheme is that many exceptions arise. Some regions are organized in a relatively simple fashion, whereas in others, like the cerebral cortex, there are many key factors to consider. Even more confusing, knowledge about different factors is often incomplete or lacking altogether. The interaction between morphology and molecular expression unavoidably adds a further level of complexity, because in neurons proteins may be expressed not only in the soma but in distant axonal or dendritic compartments. Another problem is that RNA-seq can show that a gene is expressed, but does not guarantee that it produces a protein.

Any nomenclature format must also take account of the fact that a given property may not always be present. As will be discussed, current research is showing that the properties that define a neuron are dependent on many dynamic factors, reflecting different functional states that can include differences in gene expression. These can be regarded in sum as the context within which a given name is applied. The solution here is to include the contextual factors in the name, so that the name is transparent in the context of other properties. We will discuss examples later in the multimodal cells of the cerebral cortex.

Including all of these factors in completely spelled out form can become cumbersome. It can be useful therefore to use abbreviations. This is already being done for a number of factors, such as cortical area and neuropeptides (M1 for primary motor area, SOM for somatostatin, etc.). We generally use abbreviations only when traditionally established.

## Principles for a Systematic Neuron Terminology

In general, it may be useful to distinguish three conceptual levels of description: a common term (the label), a definition (a combination of key properties), and additional features (other non-essential properties). Consider for example the entity associated with the term “neuronal dendrite.” The NeuroLex definition is “A protoplasmic process of a neuron that receives and integrates signals from other neurons and conveys the resulting signal to the cell body.” This definition identifies the unique set of necessary and sufficient properties of dendrites. Additional descriptive properties may be useful to identify a dendrite (branch length, tapering, microtubule-associated protein expression, presence of post-synaptic densities etc.), but are not part of the definition. The problem with existing common neuron names is that proper definitions are lacking in the vast majority of cases, and the label often reflects non-essential descriptive properties (such as “bistratified” or “horizontal”).

A comprehensive attempt was made by the “Petilla Conference” to list the most common properties used to classify the inhibitory interneurons of the cerebral cortex, all the way to quantitative measurements of cell processes and features (Petilla Interneuron Nomenclature Group [PING] et al., 2008). In practice, the investigator seldom has access to all of these measurements and properties. A practical approach is therefore needed that provides a format for including properties that are usually available, especially ones that are the most relevant to critical functions that may be under investigation. That will be the approach taken here. Issues related to formal ontology are covered by authors cited previously.

Synapses are a cardinal feature of neurons, expressing and determining their interactions with other neurons and cells to generate the behavior of the organism (Shepherd, 2004). A systematic approach to describing the synaptic organization of neurons is therefore a logical basis for formulating a terminology that defines a given neuron type in terms of its function. It needs to start with the morphology of the neuron, the classical basis for neuron names. Especially for the cortex, it must include localization in relation to lamination and to projections, as well as potential connectivity (with input/output directionality) within the circuit (Ascoli and Wheeler, 2016; Rees et al., 2017).

This classical unimodal anatomical approach based on structure, however, is not enough in a molecular era. One must be able to add other properties that may be judged as essential for the identity and specific function of that neuron type. These may include, for example, biophysical properties, such as ionic currents critically involved in distinct functional features, such as action potential generation and firing patterns. They must include synaptic pharmacology, such as neurotransmitter receptors and neurotransmitters or neuromodulators released, and they must include data on cell markers identified by antibody staining and, increasingly, data on gene expression.

How detailed should the morphology be in identifying a neuron type? The Petilla terminology provided the option to account for quantitative measurements of dendritic branch sizes and branching patterns. NeuronDB introduced the concept of canonical dendritic branching types in which these branching details are considered not necessary to the basic identity of the neuron. Similarly, dendritic spines are important subcellular structures for synaptic connections in certain types of neurons, for example cerebellar Purkinje cells, olfactory granule cells, neostriatal medium spiny cells, and cortical pyramidal cells. However, except for neostriatal medium spiny cells, which are otherwise defined by their connectivity and output sites, neuron identity can presently be made without relying on the spines. This situation is likely to change as more is learned about the critical properties of these important structures.

All of this needs to be provided within a framework of overall data about the subject: we assume therefore that each full name technically would begin by indicating the species (and where applicable the strain), gender, and age (Table 2). To simplify, in this review we will focus on the terminology for mouse (strain unspecified, gender unspecified, and age adolescent to mature adult).

**Table 2:** Basic features that apply to all neuron nomenclatures.

a. Species	mouse
b. Strain	see e.g., jax.org/mouse-search and purl.obolibrary.org/obo/NCBITaxon_10090
c. Gender	m/f
d. Age	embryo, newborn, young, adolescent, adult, old


*The embryo phase, with its many stages of neurogenesis and migration underlying early development, warrants its own expanded nomenclature (cf Figure 9).*

This may seem to be a large amount of data to include in a name, which is why names and properties have until now been listed separately in NeuronDB, NeuroLex Neuron, Hippocampome.org, BAMS, NeuroMorpho.Org, and most other databases.

## Application of Principles to Specific Examples

We assume a consensus of common names as contained in the NIF, BAMS, synaptic organization of well-studied regions (Shepherd, 2004) and microcircuits in over 50 brain regions (Shepherd and Grillner, 2018). We start with this traditional name that captures the essential features of the distinct morphology of neurons, and add to it the combination of properties that defines a unique neuron type. We focus on methods of specifying names of neurons that identify neurons in research reports and databases containing information derived from or supplemental to those reports. Every neuron name has the flexibility of being augmented (to select subsets) or diminished (selecting supersets) to match the study. It will be for future neuroscientists to continue to work toward a consensus on the ontology of the names for unambiguous retrieval by arbitrarily combinatorial digital search.

## Importance of Region

Central nervous systems are characteristically organized in terms of regions where neurons interact with each other, and pathways which carry connections between the regions. We recognize that whereas for most of us it is sufficient in practice to refer to a standard text or atlas (e.g., Paxinos and Franklin, 2001; Allen Mouse Brain Atlas^3^), many regional boundaries are controversial. When the region is a part of the name, as proposed here, it makes it that much more important. Many feel that anatomical boundaries are better replaced by three-dimensional coordinates in a consensus brains atlas. For present purposes we assume the reader will identify the region from their own atlas. See also the discussion of relating neurons to regions in Ecker et al. (2017).

Two issues are important to recognize here. The first is that the required granularity with which anatomical regions are delineated is hardly agreed upon in the community. Even for an intensely scrutinized neural system such as the hippocampal formation, the most up-to-date version of the Common Coordinate Framework (CCF) of the Allen Mouse Brain Atlas (arguably the most widely accepted freely available scholarly resource for this purpose) is rather non-uniform: it divides the dentate gyrus in three layers (molecular, granular, and polymorphic), but it leaves areas CA3 and CA1 undivided. The dentate laminar distinction is justified because different neuron types reside in different layers: granule cells in granular layer, mossy cells in hilus (polymorphic), and distinct types of GABAergic interneurons in each of the three parcels. Moreover, this lamination reflects the input/output organization of the principal cells and thus of the whole local circuit, as the axons and dendrites of the granule cells extend into the molecular layer and the hilus, respectively. These same reasons, however, also apply to areas CA3 and CA1, which should therefore be divided into layers as well. At the same time, many argue that the dentate gyrus molecular layer should be subdivided further since “semilunar” granule cells are only found in the inner one-third (Williams et al., 2007) while neurogliaform cells are only found in the outer two-thirds (Armstrong et al., 2011). Meanwhile, at the circuit level, the input from the entorhinal cortex is exclusively limited to the outer molecular layer while the feedback from hilus is limited to the inner molecular layer.

The second issue is that the order of priority in dividing a neural system into regions is not always straightforward. For example, different researchers may wish to specify the location of a pyramidal cell within the principal layer of area CA1 by depth (deep vs. superficial), by transversal position (closer to CA2 or to subiculum), or along longitudinal axis (from septal to temporal). Distinct properties appear to be organized along those dimensions, such as (among several others): phase-locking to distinct rhythms (Valero et al., 2015), separate axonal targeting (Tamamaki and Nojyo, 1990), and differential gene expression (Cembrowski et al., 2016), respectively. Thus, depending on the focus of a study, one and the same CA1 pyramidal cell might be described as placed “in the superficial layer,” “near the subicular border,” or “towards the septal pole,” but these descriptors are complementary and not indicative of mutually exclusive types.

Within a region (identified with the above caveats) are a set of different types of neurons that are characteristic for that region. The first basic rule for terminology of neuron types recognizes that they are extraordinarily diverse across all regions, and that each type is usually unique in its soma-dendritic morphology, which is traditionally the main criterion for naming. The first requirement for the name is therefore that it begin with the region in which the neuron is located. This is in line with much of common usage. For example, we refer to a “cerebellar Purkinje cell” to discriminate between it and a cardiac Purkinje cell, though we can drop the adjective when the distinction is clear. Similarly we refer to a “cerebellar granule cell” to discriminate between it and quite different neurons named granule cells in other regions. This enabled the NIF NeuroLex list of over three hundred names to be organized coherently on an easily searchable regional basis. Similar considerations will apply to neuron databases using the proposed format.

## Nuclear Regions: Neuron Names Follow Parent-Child Format

We begin by considering examples of brain regions for applying the proposed terminology format in order to test the general validity of the approach. Regions may vary from simple, in which the set of different neurons is relatively homogeneous throughout a region, to complex, containing subregions, laminae, clusters, etc.; these delineations are not always agreed upon, as mentioned above. The simplest case is often called a “nucleus.” Examples are the ventral horn of the spinal cord containing the motor neurons projecting to the muscles; the caudate and putamen of the neostriatum; and the many “nuclei” that characterize the central nervous system of the avian brain. This homogeneity implies that the entire region is organized to generate a specific set of functions, combining that with operations on its inputs to send outputs to other regions.

### Spinal Cord

**FIGURE 1:**
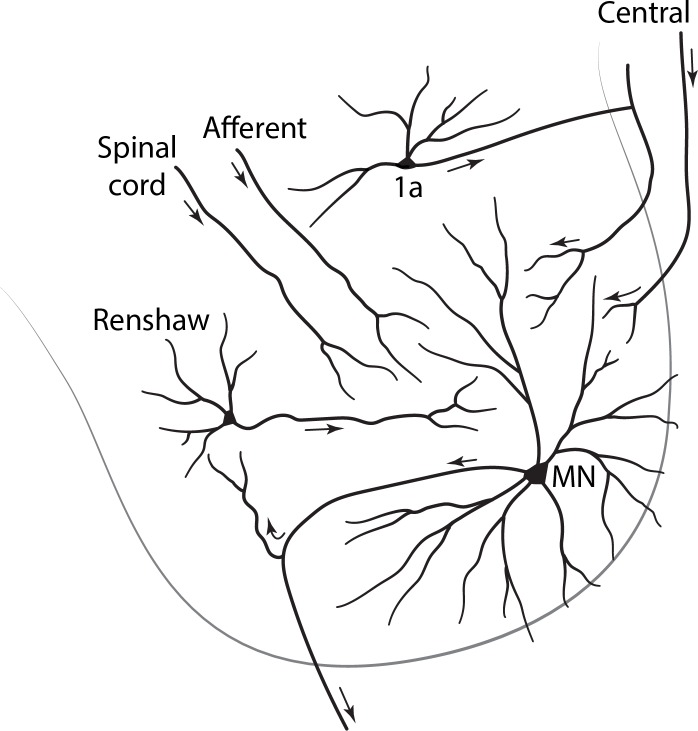
Cross section of the lumbar spinal cord, showing in the ventral horn a principal neuron (alpha motor neuron: MN) with long motor axon to a distant target, a skeletal muscle; and interneurons with short axons that remain within their region of origin.

As an example we take the ventral horn of the spinal cord. The cord occurs in four main groups of segments: cervical, thoracic, lumbar and sacral. The lumbar ventral horn contains two types of neurons: large cells, motor neurons, which have long axons that innervate the skeletal muscles and muscle spindles, and small cells, which have short axons that stay within the anterior horn (Figure 1). Golgi was the first to differentiate between long axon cells and short axon cells, and that distinction remains fundamental. The current convention is to call those with long axons principal cells and those with short axons interneurons. In the NeuronDB list, the nomenclature is simplified by indicating principal neurons in dark green, and interneurons slightly indented and in light green. For present purposes, in order to make the terminology as efficient as possible, any neuron type not labeled interneuron is assumed to be a principal neuron without adding “principal (‘P’),” unless it is needed for clarity.

Traditionally “interneuron (‘int, INT’)” has been used rather loosely, sometimes referring to any cell that connects to other cells, but it is much more useful to use this term to designate the type of cell whose direct actions are entirely local. This will include a cell that lacks an axon as well (as in the retina and olfactory bulb). We note that a short axon cell can have inputs coming from long axons, and long axon cells may give off collaterals that stay within the local region. We will also note later that interneurons have been found whose axons connect local arborizations in different regions.

With these distinctions, we have “Spinal cord ventral horn motor neuron” and “Spinal cord ventral horn interneuron” as traditional names for these two types of cell in the vertebrate spinal cord. However, because the spinal cord contains several regions, we need to clarify which region contains these cells. We thus have expanded terms for region, subregion, and axon type as: “Spinal cord lumbar ventral horn motor neuron” and “Spinal cord lumbar ventral horn interneuron” (Figure 1). Note how the inversion of the adjectival “lumbar” is necessary to keep all that follows under the common term “Spinal cord.”

Ventral horn motor neurons come in two sizes with different connectivity: large alpha and smaller gamma. Interneurons may be of several types: Renshaw, Ia, Ib, etc. The controlling rule for adding these to the names is that in order to maintain the alphabetical order they must be modifiers of the main types, i.e., they must satisfy a parent-child relation, giving “Spinal cord lumbar ventral horn motor neuron alpha” and “Spinal cord lumbar ventral horn interneuron Renshaw.” These examples are fully consistent with the format used in NeuronDB and NeuroLex Neuron.

The terminology builds directly on the chapter on “Spinal Cord” by Robert Burke in *The Synaptic Organization of the Brain*, ed. 5 (2004). The structured terminology ensures that the neurons are listed together in logical parent-child relations for easy visual review. Note that the principal neurons are differentiated on the basis of size, but also more distinctively on connectivity to completely different targets, alpha motor neurons to skeletal muscle and gamma motor neurons to muscle spindles. We will see that morphology and connectivity will vie for priority in defining neurons in many regions; together, they are in fact usually enough to specifically define the cell type. In ventral horn neurons, differentiating between motor neurons and interneurons, and between subtypes of each, is relatively easily possible because of their relation to specific reflex pathways that experimentally can be differentially activated.

With regard to the functional properties in our classification, alpha motor neurons express RNA for *Err3* (Friese et al., 2009); firing may be adapting or non-adapting, and acetylcholine (ACh) is the transmitter, which is excitatory at skeletal (skel) muscle endplates. Renshaw cells are known to be bursting, releasing glycine which is inhibitory to motor neurons. More complete names for the alpha motor neuron and the Renshaw interneuron are thus (*Err3*: Friese et al., 2009):

**Table:** 

Region	Subreg	Layer	Conn	Trad. Name
Spinal cord	lumbar	VH	skel	MN alpha
Spinal cord	lumbar	VH	INT	Renshaw
Genes	Peptides	Physiol	Trans
*Err3*		adapt	ACh
		burst	GLY

If data on properties is missing, as for genes and peptides in this case, the category may be omitted and the name can be written in succinct form; the abbreviations are standard and therefore need no explanation:

Spinal cord lumbar VH INT Renshaw burst GLY

### Neostriatum

**FIGURE 2:**
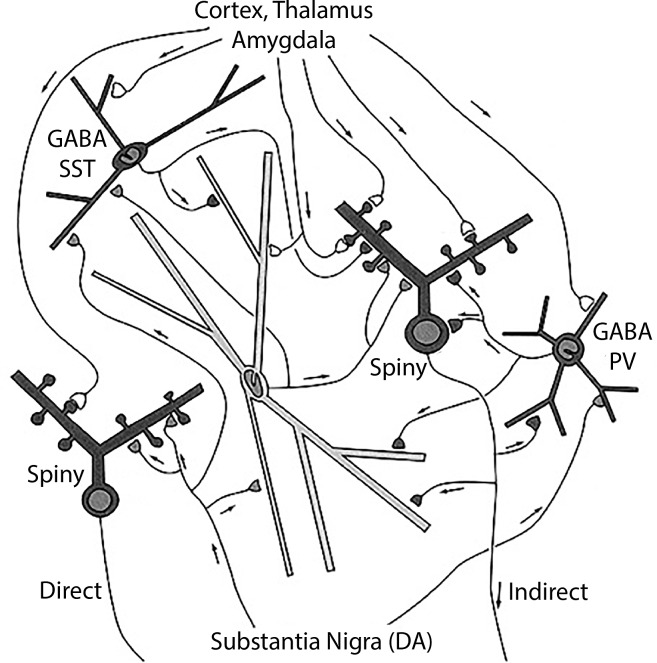
Neostriatum, showing the direct and indirect principal neuron projections to the globus pallidus interna by the medium spiny neurons, and the several types of interneurons, including the large cholinergic interneuron [see text, adapted from Wilson (2004)].

The neostriatum consists of the caudate and putamen nuclei, which are very similar in neuronal constituents (Doig and Bolam, 2018): one set of output (principal) neurons, the medium spiny neuron (spiny), is divided into two subtypes depending on the external target. Some (spiny indirect) connect indirectly to the globus pallidus pars interna through a relay in the pars externa, while others (spiny direct) connect directly to the pars interna (Figure 2). Within the neostriatum the main type of interneuron is the large cholinergic interneuron. In traditional terms: “neostriatal direct medium spiny neuron”; “neostriatal indirect medium spiny neuron”; and “neostriatal large cholinergic interneuron,” terminology consistent with NeuronDB and NeuroLex Neuron. In contrast to the ventral horn, the medium spiny neurons are relatively homogeneous in appearance, so differentiation between direct and indirect subtypes requires further physiological or molecular approaches (see below).

In this context, a problem arises if an experimental study is carried out reporting properties of medium spiny neurons in which the differentiation between direct and indirect connectivity is not known. Where do the properties get assigned? One possibility is a third category of “Undifferentiated medium spiny neuron.” Another possibility is to enter the property into both types, with an asterisk or other sign that indicates differentiation unknown.

The neostriatum has also been characterized in terms of an organization of the medium spiny neurons into patches (striosomes: strio) within a continuous matrix (Graybiel, 2018). The medium spiny neurons have both direct and indirect projections from these entities. For some purposes, striosome and matrix locations of the cell bodies may be more important for the identities of the medium spiny neurons under investigation. A cell type with a given soma and dendritic morphology may therefore have a multiple identity depending on whether it is being characterized by its soma location or its axonal projections. This multiple identity is contained within the hierarchically organized name.

Data on genes include an early marker *DARPP-32* (Ouimet and Greengard, 1990) and differential expression of dopamine receptors 1 and 2 in direct and indirect medium spiny neurons, respectively. Thus, at present, we have, as examples for database entries, for a principal neuron and the large cholinergic interneuron (choline acetyltransferase: CHAT):

**Table:** 

Region	Subreg	Layer	Conn	Trad. Name
Striatum	caudate	strio	direct	med spiny
Striatum	caudate	strio	INT	cholinergic
Genes	Peptides	Physiol	Trans
*Ppp1r1b, Drd1*		non-adapt	GABA
*CHAT*		burst	ACh

## Application to Cortical Neurons: The Challenge of Cortical Layers and Cortical Areas

The approach thus far indicates that developing a systematic nomenclature for even apparently simple brain regions has many issues; nevertheless, we can develop and refine methods which can then apply to the complexities of cortical regions. In the same way, an approach through simpler types of cortex may also be effective. In recent years there has been increasing interest in the evolution from three-layer cortex, as exemplified in the olfactory and hippocampal cortices, to six-layer neocortex (Shepherd, 2011; Aboitiz and Montiel, 2015; Brunjes and Osterberg, 2015; Fournier et al., 2015; Luzzati, 2015; Diodato et al., 2016; Rowe and Shepherd, 2016; Klingler, 2017; Naumann and Laurent, 2017; Shepherd and Rowe, 2017). A nomenclature for cortical neurons should therefore be consistent with an evolutionary perspective. An exciting possibility is that the proposed approach to the nomenclature could ultimately reflect, and give insight into, the evolutionary processes that formed the neocortex and its neurons. For this purpose, the cortex of the present day turtle has become of interest as providing a lens into the forerunner of the earliest mammalian neocortex over 200 million years ago. We diverge to consider it briefly.

### Turtle Dorsal Cortex

**FIGURE 3:**
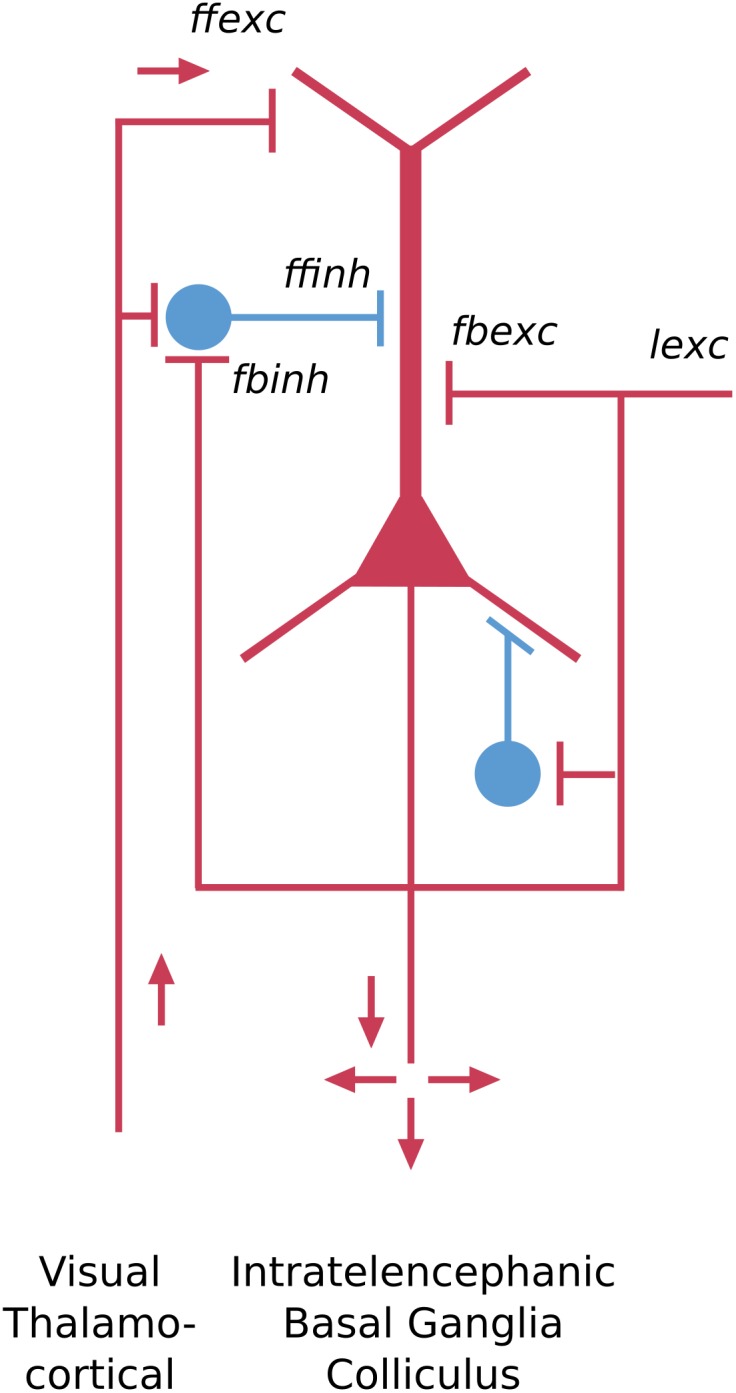
Basic pyramidal cell (principal cell) forms the cortical circuit module of three-layer cortex in the turtle dorsal cortex. Red indicates excitatory, blue indicates inhibitory cell. *ffexc*, feedforward excitatory; *fbexc*, feedback excitatory; *lexc*, lateral excitatory; *ffinh*, feedforward inhibition; *fbinh*, feedback inhibition [Adapted from Shepherd and Rowe (2017)].

The dorsal cortex of the turtle has a single layer of pyramidal cells. As the name suggests, it lies dorsally, between the lateral olfactory cortex and the medial hippocampal cortex (ten Donkelaar, 1998). It is referred to as a “three-layer” cortex: a layer of pyramidal neuron bodies, between a deep layer of fibers and a superficial layer of dendrites, interneurons, and fibers (Smith et al., 1980; Kriegstein and Connors, 1986). As shown in Figure 3, the “pyramidal” name refers to the shape of the cell body. The cell has basal dendrites and a single apical dendrite, both covered in dendritic spines where excitatory synapses are made. The axon gives off collaterals which provide for excitation of itself and neighboring cells, and excitation of interneurons that provide for feedback and lateral inhibition of itself and neighboring cells. The axon then exits to target other cortical regions as well as the basal ganglia. We adopt current terminology for the neocortex in referring to this type of connectivity remaining mainly within the forebrain cortex and basal ganglia as “intratelencephalic (IT)” (Reiner et al., 2010; Harris and Shepherd, 2015; Shepherd and Rowe, 2017). There is also a projection to the superior colliculus.

The key features of this neuron type include the pyramidal-shaped cell body, the basal and apical dendrites, their dendritic spines, the recurrent and axon collaterals providing for feedback and lateral excitation and inhibition, and the projections to other “intratelencephalic” regions (see Shepherd, 2011). From an evolutionary perspective, this cell type can be traced back past the reptiles to the earliest vertebrate ancestors in fish and lamprey (Suryanarayana et al., 2017), where it appears in more generalized forms.

Applying the nomenclature rules thus far, assuming turtle instead of mouse for species, this neuron would be designated in full as:

**Table:** 

Species	Region	Subregion	Connect
turtle	forebrain	cortex dorsal	IT
Name	Physiol	Trans
pyramidal	non-adapting	GLU

Further data on gene expression and cell markers are under investigation. Comparisons with simple piriform cortex and layers of neocortex are discussed below.

### Piriform “Olfactory” Cortex

**FIGURE 4:**
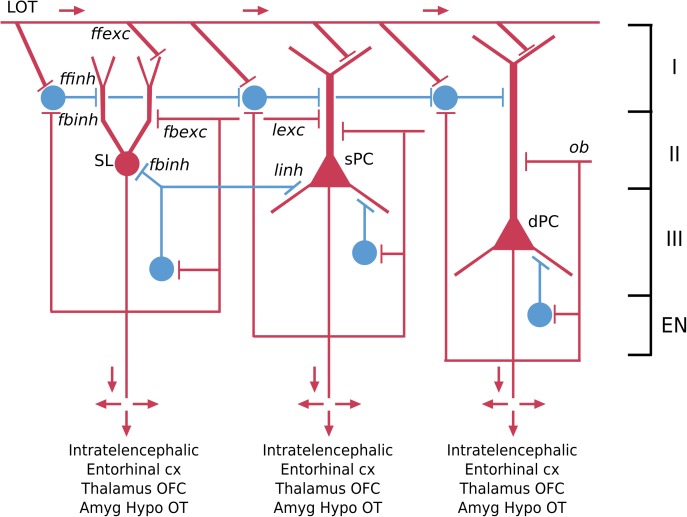
Basic pyramidal cell (principal cell) circuit modules of the piriform (olfactory) cortex. Red indicates excitatory neurons, blue indicates inhibitory neurons. SL, semilunar pyramidal cell; sPC, superficial pyramidal cell; dPC, deep pyramidal cell [Adapted from Shepherd and Rowe (2017)].

The piriform cortex is often referred to as olfactory cortex because it receives the output fibers of the olfactory bulb, and is an essential link to the neocortex where olfactory perception arises. This cortex arose in fish, amphibians and reptiles. Like dorsal cortex, it is usually referred to as a “three-layer” cortex. Closer observation shows that the cellular layer is actually composed of three distinct types of principal neuron in three layers: a most superficial layer of pyramidal semilunar (SL) cells; a middle layer of superficial (spc) pyramidal cells; and a layer of deep (dpc) pyramidal cells (Neville and Haberly, 2004; Wilson and Barkai, 2018) (see Figure 4). The piriform cortex is also divided into anterior and posterior parts.

The semilunar cell, though lacking basal dendrites and a single apical dendrite, is usually classified as a variant of a pyramidal neuron because of its extensive association fiber connections within the olfactory cortex, with the olfactory bulb and with the endopiriform (EN) nucleus, as well as its glutamate transmitter. Applying the nomenclature rules, the semilunar cell in a mammal would thus be designated in full:

**Table:** 

Region	Subreg	Layer	Conn	Trad. Name
Piriform	anterior	supfl	IT	semilunar
Genes	Peptides	Physiol	Trans
		non-adapt	GLU

Similarly, a second type, the superficial pyramidal cell, also has axon collaterals within the olfactory cortex, providing both the excitatory and, through an interneuron, inhibitory feedback. We also have data on functional properties. RNA-seq shows expression of several genes such as cux1 in this layer, but it is not at the single cell level (Brunjes and Osterberg, 2015). Action potential firing tends to be non-adapting and the neurotransmitter is glutamate. The name would thus be:

**Table:** 

piriform	anterior	supfl	IT	pyramidal	Cux1
non-adapt	GLU

Similar rules apply to the deep pyramidal neuron and the pyramidal neurons in the posterior cortex.

The corresponding interneurons are classified as superficial, middle and deep (see Figure 4). Their full names follow the usual rule: for example:

**Table:** 

piriform	anterior	supfl	INT	superficial
burst	GABA

### Hippocampus

A third type of three-layer cortex in the mouse is the hippocampus. The olfactory cortex has provided examples of principal neurons arranged in different layers (Figure 4); the hippocampus particularly provides examples of principal neurons organized into distinctly different areas.

**FIGURE 5:**
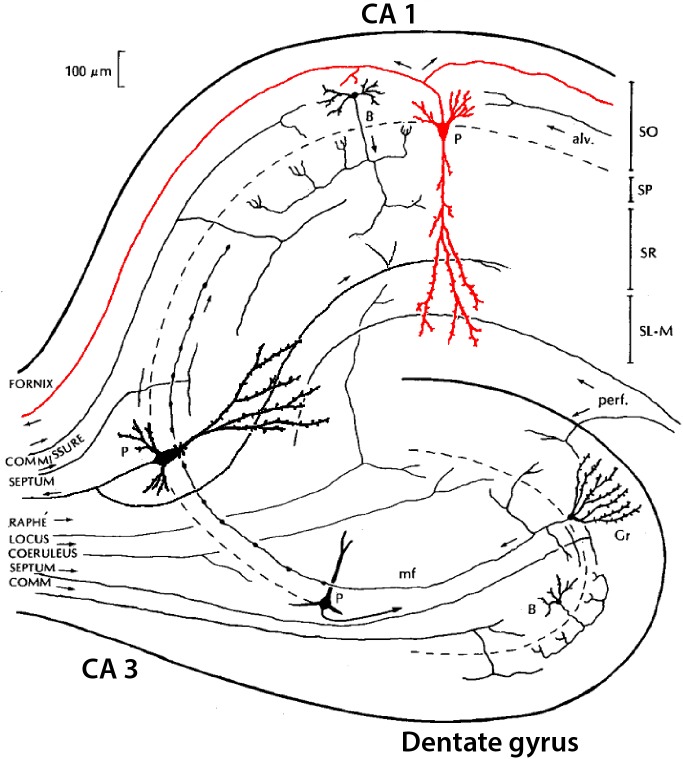
Simplified summary of neurons in the hippocampus, showing the three main subregions: dentate gyrus (DG), CA3, and CA1, with their three main principal neurons: granule cell (Gr), and pyramidal (P) cells in CA3 and CA1. Examples of interneurons are shown for basket (B) cells in dentate gyrus and CA1 [Adapted from NeuronDB: (https://senselab.med.yale.edu/FunctionalConnectomeDB/realisticdiagram/diagram.py?id=154765)].

Classical studies identified a region in the temporal lobe that had a coiled structure remindful of a seahorse (hippo, horse; campus, monster) and a ram’s horn (cornu ammonis: CA). The hippocampal complex is divided into a dentate gyrus and the hippocampus proper, the latter of which is further divided, in rodents, into three areas (CA1-3). We focus here on neurons of the dentate, CA3, and CA1 (see Figure 5). Classically, the cell bodies all appear to be localized in a single layer within each area (granule cell layer in the dentate; pyramidal cell layer in CA3 and CA1). Some studies find it useful to separate further CA1 pyramidal cells into superficial and deep subtypes (see Slomianka et al., 2011; Valero and de la Prida, 2018). The dentate principal neurons are called granule cells, a name which arose only because they appeared very small in early microscopic studies; they have no necessary relation to granule cells in other parts of the nervous system. The parent-child format of region-name ensures there is no confusion. They have bushy spiny dendrites extending into a molecular layer where they receive input from stellate neurons in the medial and lateral entorhinal cortices. Their mossy fiber (MF) axon extends in a curving arc through the polymorphic layer (also known as the hilus) to terminate in large “mossy”-appearing terminals onto specialized sites (thorny excrescences) on proximal apical dendrites of pyramidal cells in area CA3. The CA3 pyramidal neuron in turn projects its axon through “Schaffer collaterals” mainly to the mid-apical dendrites of CA1 pyramidal neurons. CA1 pyramidal neurons project to the subiculum. All three of these principal neurons release glutamate as their neurotransmitter and are excitatory.

Recent studies are beginning to provide evidence for the richness of gene expression in hippocampal neurons. Examples for the principal neurons from Cembrowski et al. (2016) can be summarized in the names by following the canonical “three-synapse circuit,” dentate to CA3 to CA1:

**Table:** 

Region	Subreg	Layer	Conn	Trad. Name
Hippocampus	dentate	gran	MF	granule
Hippocampus	CA3	pyr	Sc	pyramidal
Hippocampus	CA1	pyr	sub	pyramidal
Genes	Peptides	Physiol	Trans
*Math-2, Tox3*		non-adapt	GLU
*Math-2, Coch*		non-adapt	GLU
*Math-2, Wfs1*		non-adapt	GLU

Similar considerations apply to names for the CA2 principal neurons.

### The Problem of Interneurons

With regard to interneurons, a common type found in all hippocampal areas is the basket cell, with long superficial and deep dendrites, and an axon that innervates the soma and nearby dendritic shafts. The common name for this cell in the dentate gyrus is therefore “hippocampus dentate interneuron basket cell.” In CA3, a basket cell interneuron would be “hippocampus CA3 interneuron basket cell,” and similarly for a basket cell in CA1. As in the rest of the hippocampus and neocortex, two mutually exclusive types of morphologically identified GABAergic basket cells are clearly distinguished in the granule layer of the dentate: those expressing the calcium-binding protein parvalbumin (PV), which are fast-spiking, and those expressing the neuropeptide cholecystokinin (CCK), which are regular-spiking. Their formatted names according to the current proposal would then be:

**Table:** 

Region	Subreg	Layer	Conn	Trad. Name
Hippocampus	dentate	gran	INT	basket
Hippocampus	dentate	gran	INT	basket
Genes	Peptides	Physiol	Trans
*Pvalb*		fast-spk	GABA
	CCK	reg-spk	GABA

Interneurons in the hippocampus, however, are not simple. Somogyi and Klausberger (2018) have reviewed evidence indicating 28 different interneuron types across the dentate, CA3 and CA1 (see their **Table 17.1**), but even this is seen by themselves as an over-simplification. For instance, distilling a rich literature spanning over two decades (Freund and Buzsáki, 1996; Pelkey et al., 2017), Hippocampome.org reports experimental evidence for as many as 71 distinct interneurons in these three areas, based on the laminar location of their axons and dendrites, the specificity of their post-synaptic targets, and clearly distinct combinations of molecular and physiological properties.

This overwhelming number of interneuron types presents several problems that are special for the nervous system and its cellular nomenclature. First is relating the neuron types to many genes and proteins and other cell markers. Second is to include their functional properties. And third is to incorporate them into the neuron nomenclature. Although it may seem that this is a problem that must somehow be minimized, this large number must rather be telling us something very important about the function of the hippocampus. Since the function of the hippocampus is crucial for understanding mechanisms of episodic memory as well as of spatial navigation and learning, the different types of interneurons are obviously critical to that understanding. This makes it all the more important to provide an efficient nomenclature system to facilitate studies at whatever level of detail is needed.

A solution to providing this support within the context of the larger nomenclature database is to compartmentalize it as a knowledge base of its own, and this has been the approach pursued by Hippocampome.org (Wheeler et al., 2015), which contains the full details of all interneuron types currently identified and is continuously updated with further types that may be revealed by future research. A start toward similar types of specialized databases for highly complex neuronal populations in the retina and the neocortex has been made in NeuronDB and in collaboration with NeuroLex Neuron (Larson and Martone, 2013). For general purposes, the main nomenclature database will include the key principal neurons and such interneurons that have particular importance for functions of general interest, as indicated above, such as generation of theta waves, long-term memory, and spatial orientation and learning.

Hippocampal interneurons are revealing yet a further complexity with neuron identities and nomenclature. In a study of transcriptomes of many hippocampal inhibitory cells, Harris et al. (2018) confirmed the presence of discrete classes, but also cells that show continuous variation in gene expression. As discussed above (see Cembrowski et al., 2016), this can mean that neuron classes based on gene expression may vary continuously with space or with activity states of the neurons.

We have focused on the adult mouse, but much interest is directed toward development for the insights it can give into how cortical neuron diversity is established (Wamsley and Fishell, 2017). Evidence is now rapidly accumulating on the subsets of transcription and related factors responsible for determining neuron types. We will return to this question in discussing neocortex below.

## Terminology for Neocortical Neurons

The neocortex is believed to have arisen in the earliest mammals around 250 mya, combining features of the three-layer olfactory cortex and reptilian dorsal cortex to form the characteristic six layers in the adult (Shepherd, 2011; Aboitiz and Montiel, 2015; Brunjes and Osterberg, 2015; Fournier et al., 2015; Luzzati, 2015; Diodato et al., 2016; Rowe and Shepherd, 2016; Klingler, 2017; Naumann and Laurent, 2017; Shepherd and Rowe, 2017). At the beginning, the neocortex was a small part of the forebrain cortex, which was dominated by a large olfactory area (Molnar et al., 2014). During mammalian evolution the olfactory area continued to dominate in the opossums, while the neocortical area expanded greatly in most other species. Thus arose the fundamental forces that formed the expanded neocortex: the multiple intracortical layers of neurons and fibers; the multiple regions reflecting differences in the layers of neurons and fibers; and the multiple input and output connections unique for the neurons of each region.

We apply the same nomenclature rules used for other parts of the nervous system, focusing on the adult mouse. No major differences in cortical regions have been reported on the basis of mouse strain or gender, so this will be unspecified.

### Principal Neurons of the Neocortex

Our nomenclature will begin as usual with the region in which it is located. There are some 42 regions in the mouse neocortex (Paxinos and Franklin, 2001; Allen Mouse Brain Atlas) (over 180 in human, another reason to begin with the mouse). Among these regions, one of the most easily recognized is the primary motor area (MOp or M1), defined as containing the neurons whose axons project directly into the pyramidal tract. The name thus begins with “Neocortex M1.”

**FIGURE 6:**
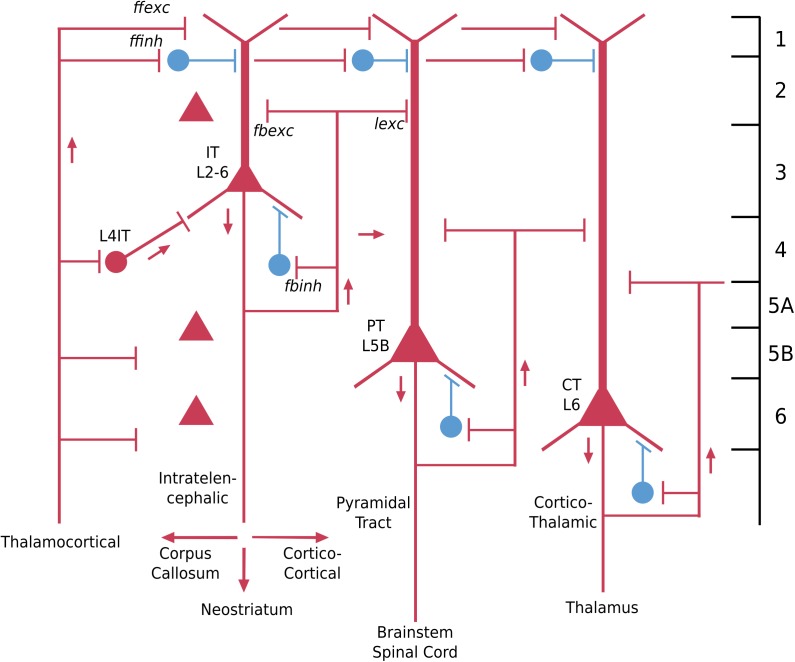
Basic pyramidal cell (principal neuron) types (in red) in the adult neocortex in relation to the six layers and to the three connectivity types: intratelencephalic (IT), pyramidal tract (PT) and corticothalamic (CT). Interneuron examples (in blue) are superficial cells in layer 1, and basket-cell types in relation to pyramidal cell bodies. Red color indicates excitatory neurons, blue indicates inhibitory neurons [Adapted from Shepherd and Rowe (2017)].

In the neocortex the basic cellular building block is the same as for other cortical regions we have discussed: the pyramidal neuron basic module, with basal and apical dendrites and recurrent and lateral excitatory and inhibitory feedback (Pyr in Figure 6). Since there are five layers containing pyramidal neurons (layer 1 lacks them), a traditional approach has been to name the pyramidal neurons in relation to the layer containing their cell bodies, which may be summarized as (Pyr L2/6). This has the advantage of relating to the cells the investigator sees under the microscope, but gives little information on their structural or functional significance.

A better alternative from that point of view is in terms of the hodology, the connectivity. In this approach, three basic connectivity profiles of pyramidal cells have been identified (reviewed in Harris and Shepherd, 2015). Some pyramidal cells have axons whose connections are entirely within the neocortex or the basal ganglia just under it; they are called “intratelencephalic” (IT) (“within the forebrain”). These are equivalent to all of the three-layer cortices we have discussed, whose output axons also are confined within the forebrain. A second type is the pyramidal tract “PT” neuron, whose axon descends to carry output from the neocortex to the brain stem and in some species all the way into the spinal cord. The third type is the cortico-thalamic (“CT”) cell, which as the name implies, connects to the thalamus, in a way that completes the loop from thalamo-cortical cells to the cortex.

These three types have different relations to the layers. IT cells can be found in all layers from L2/6, although especially in superficial layers 2/3. PT cells are found specifically in L5b, whereas CT cells are found in L6. It should be emphasized that a layer may contain cells with different axonal targets (cf Thomson, 2010). This approach to classifying neocortical neurons is illustrated in Figure 6. Thus the traditional name for intratelencephalic neurons in primary motor cortex is: “neocortex M1 L2/5 IT pyramidal cell.” Adding known functional properties (N. Sestan, personal communication for genes) gives, respectively:

**Table:** 

Region	Subreg	Layer	Conn	Trad. Name
Neocortex	M1	L2/6	IT	pyramidal
Neocortex	M1	L5b	PT	pyramidal
Neocortex	M1	L6	CT	pyramidal
Genes	Peptides	Physiol	Trans
*Cux1, Satb2*		non-adapt	GLU
*Sox5-Fezf2*		non-adapt	GLU
*Zfpm2*		non-adapt	GLU

Note that cux1 is also expressed in olfactory cortex (see above). Identifying genetic or molecular markers at the single cell level for these principal neurons is now beginning (Shibata et al., 2015; Gillespie et al., 2018; Gouwens et al., 2018; Tasic et al., 2018), as discussed below.

### Interneurons of the Neocortex

We have seen that in the case of the hippocampus, there is to a certain extent only a single type of pyramidal neuron for each area CA1-3, but many types of interneurons. The situation is similar in the neocortex where, although numerically 80% of the cells are glutamatergic pyramidal cells and 20% gabaergic interneurons, so far a greater diversity has been discovered in interneurons. From a circuit viewpoint, neocortical interneurons can be divided into three main families: perisomatic (whose axon targets largely the cell body, the proximal dendrites, and the axonal initial segment), which are mainly responsible for controlling the output of pyramidal cells; axo-dendritic (largely targeting the intermediate and distal dendrites), which are responsible for controlling the input to pyramidal cells; and interneuron-specific (mainly targeting other gabaergic cells), which are responsible for controlling disinhibition. As an example of the first family, there are chandelier cells connecting to pyramidal neurons in most layers; parvalbumin (*Pvalb*) has become a marker for this cell type, so we can incorporate that in the name: thus “neocortex M1 L1/6 interneuron chandelier PV cell.” Similarly, Martinotti cell containing somatostatin (an instance of the second interneuron family) may be named: “neocortex M1 L2/6 interneuron Martinotti SOM cell.” A main type of the third family is the bipolar interneuron containing vasoactive intestinal peptide: “neocortex M1 L2/6 interneuron bipolar VIP cell.”

**Table:** 

neocortex	M1	L1/6	INT	chandelier
neocortex	M1	L2/6	INT	Martinotti
neocortex	M1	L2/6	INT	bipolar
*Pvalb*		burst	GABA
	SOM	burst	GABA
	VIP	burst	GABA

Note that these three types are also found in all other regions of the cortex, including visual (Jiang et al., 2015) and somatosensory (Markram et al., 2004). This emphasizes the importance of attaching each neuron type to its brain region, to track whether the cell properties and functions are similar or different across regions.

We have focused on three main types of interneurons in the neocortex, but as in the hippocampus there are many more. For example, Markram et al. (2004) described a basic set of 12 in their analysis of neuropeptides (see below). Schuman et al. (2019) describe 4 unique interneuron populations in layer 1 alone, each with unique morphology, gene expression and physiology.

Finally, the basic distinction between a principal, long-axon, cell projecting to other regions, and interneurons projecting only within their region, is beginning to yield to findings of gabaergic interneurons with long projecting axons. For example, optogenetic activation of a somatostatin-containing interneuron in mouse neocortex has been shown to modulate spike-timing through gabaergic inhibition of medium spiny output neurons in the striatum (Rock et al., 2016), a specific function controlled by a genetically defined type of interneuron in a distant cortical region. This balance between the excitatory PT and IT cells and inhibitory cortical somatostatin interneurons is believed to shape the timing of motor control output from the striatum in response to sensory stimuli. Further examples of long-range interneuron inhibition are cited in Yamawaki et al. (2019). We thus have examples of long range axon collaterals involved in local circuits and short-range axons also involved in long-range circuits. Our terminology can accommodate these variations by indicating an interneuron connecting to two or more regions as a combined interneuron and principal neuron (INT/P):

**Table:** 

Necortex	M1	L2/3	INT/P	basket	SOM
burst	GABA

### The Problem of Large Numbers of Cortical Neuron Types

In NeuroLex Neuron, a start was made (G. Shepherd, S. Larson, M. Martone and K. Rockland) to building a specialized database for neocortical neurons. This consisted, briefly, of separate sections for principal neurons and interneurons, each arranged to contain different cell types in individual layers of distinct cortical areas. In the current approach this would at least be simplified to the extent of covering only mouse. However, even then, in terms of layers, one would potentially have a minimum of IT pyramidal cells in all 5 cell layers, plus PT in layer 5b and CT in layer 6, for a total of 7 types, plus at least 3 interneuron types in each of the 6 layers, for a total of 25 neuron types in one cortical area; considering 42 areas makes a total of over 1,000 different neuron types, by layer and connectivity, in the neocortex of an adult mouse. Humans with over 180 neocortical areas must have many times more.

These numbers likely underestimate the number of neuron types in the neocortex. The IT type of pyramidal cell potentially makes connections to the ipsilateral and contralateral neocortex, and different targets within the basal ganglia (Shepherd, 2013; Harris and Shepherd, 2015). Not every cell projects in exactly the same manner to every target neuron within these regions. Left largely undetermined is how different the projections are to different combinations of cells, but recent findings in this regard potentially point to a combinatorial explosion of possibilities (Economo et al., 2018), which will likely greatly diversify the IT cell population. Similar considerations would appear to apply to PT neurons, which in aggregate project to many regions within the subcortical neuraxis; different PT neurons are known to end on different subpopulations of target neurons (Kita and Kita, 2012), greatly amplifying the combinatorial possibilities.

These many potential types might suggest that we should try to lump as many as possible together to make our database more manageable. However, a better conclusion is that this approach to neuron terminology is revealing one of the essential features of the neocortex: the uniquely large number of different neuron types, each potentially able to process information in ways that differ either slightly or radically in generating enhanced behavior of the animal.

## Adding Properties to Neuron Names: Increasing the Chain

We next discuss in more detail the multiple neuron properties that can be added to the name. In NeuronDB, NIF, Hippocampome.org, and NeuroMorpho.Org, the name for most neurons is succinct, and the properties that characterize the neuron are contained in a separate section. However, the present approach enables most characterizing properties to be incorporated into the name itself. As has been emphasized, this has two advantages: the properties of a given neuron are more obvious in the name, and in a listing of all neurons the properties are quickly accessible to search.

### Gene Expression

In some cases the properties naturally become part of the name as research correlates morphology with marker molecules; see the interneurons in the hippocampus and neocortex. This trend will strongly increase in the future, especially for gene expression in specific neuron types. Single cell PCR with RNA-seq and related methods are already adding many genes expressed in single identified neurons. In our notation scheme the gene names can be added beside the marker labels. We will discuss problems with identifying genes with high throughput RNA-seq methods, combining the genes with functional methods and neurotransmitters, and finally incorporating the most recent data into our naming format.

An advantage of including a specific expressed gene or RNA in the name is that it can then be searched for across different regions and neuron types. We have noted a study comparing gene expression between three-layer olfactory cortex and six-layer neocortex (Brunjes and Osterberg, 2015) that found expression of cux1 in the layers containing both the superficial pyramidal neurons of anterior piriform cortex and presumed IT pyramidal neurons of superficial neocortex. These findings could be incorporated into the names when established at the single cell level. This would greatly facilitate the study of gene expression and neuron identity in these two cell types, a study which may also give insight into the evolution of the neocortex as noted above.

A problem will be what to do as dozens and hundreds of genes are identified that could be added to the name. The key will be to focus on the genes that are essential to differentiating that cell’s identity. At this point there is no consensus answer. We can suggest several possibilities (see also below under Physiology). First, genes can be related to a given neuron in a separate specialized database, as noted above for the hippocampus. Second, a specific gene or several genes may be of special interest in a given context. Third, in the primary database the genes may be retrieved by hovering over a single characteristic gene to reveal all the genes expressed in that cell. This is challenge for the future.

A further problem for the whole idea of specific names for neuron types is the recent study mentioned above of hippocampal interneurons by Harris et al. (2018), which found continuous variation in classes of properties:

“A division into discrete classes, however, was not sufficient to describe the diversity of these cells, as continuous variation also occurred between and within classes. Latent factor analysis revealed that a single continuous variable could predict the expression levels of several genes, which correlated similarly with it across multiple cell types. Analysis of the genes correlating with this variable suggested it reflects a range from metabolically highly active faster-spiking cells that proximally target pyramidal cells to slower-spiking cells targeting distal dendrites or interneurons. These results elucidate the complexity of inhibitory neurons in one of the simplest cortical structures and show that characterizing these cells requires continuous modes of variation as well as discrete cell classes.”

The authors conclude:

“Our data suggest a common genetic continuum exists between and within classes, from faster-firing cells targeting principal cell somata and proximal dendrites, to slower-firing cells targeting distal dendrites or interneurons. Several classes previously described as discrete represent ranges along this continuum of gene expression.”

We will further discuss this variation in physiological properties in the next section.

### Physiology

As already indicated, incorporating physiological properties into the classification of a neuron has turned out to be surprisingly difficult. Whereas agreement on the morphology of a neuron is relatively straightforward, there is little agreement on how to include physiological properties. Many electrophysiologists argue that it cannot be done: physiological properties by their very nature are exquisitely dependent on many factors affecting the state of the recorded neuron, including age, anesthetic, animal treatment, temperature, duration of the experiment, *in vitro* recording, slice methodology, behavioral setup, solution composition, type of recording electrode, damage by the recording electrode, identification of the recorded neuron, activity due to injected current or stimulation of inputs, to name a few. Few classifications of neurons therefore include physiological properties among criteria for neuron identification.

Despite these multiple variables, many neurons do show clear types of properties that must be crucial for their function. One type of property is the basic biophysics of the cell as tested by intracellular recordings: its input resistance, membrane resistance, membrane time constant, spike half-width, after-hyperpolarization, etc. These are essential, when combined with the morphology, for constructing a model of the neuron that can simulate how it carries out its input-output operations. Such data from the publications that reported them are now available for over 70 neuron types at NeuroElectro.org, the most specialized database for electrophysiological properties. Data on membrane properties are also archived in CellPropDB for whole neurons and for neuronal compartments in NeuronDB; the models that combine these properties with the morphology are archived in ModelDB. Moreover, Hippocampome.org reports all known biophysical parameters for morphologically identified neurons in the hippocampal formation.

An important general conclusion from comparing the properties in these databases is that, in general, many properties, such as Na_t_ in axons and GLU receptors in dendrites, are found in most neurons. Differentiation of function occurs through localization of precise combinations of properties (often reflecting selective receptor subunit expression) in specific axon, soma, and dendritic compartments, as archived in NeuronDB, and demonstrated by the models in ModelDB. For most purposes the genetic basis of these properties will be reflected in the physiology; the genes responsible can be included in the name when they are relevant to a particular investigation.

**FIGURE 7:**
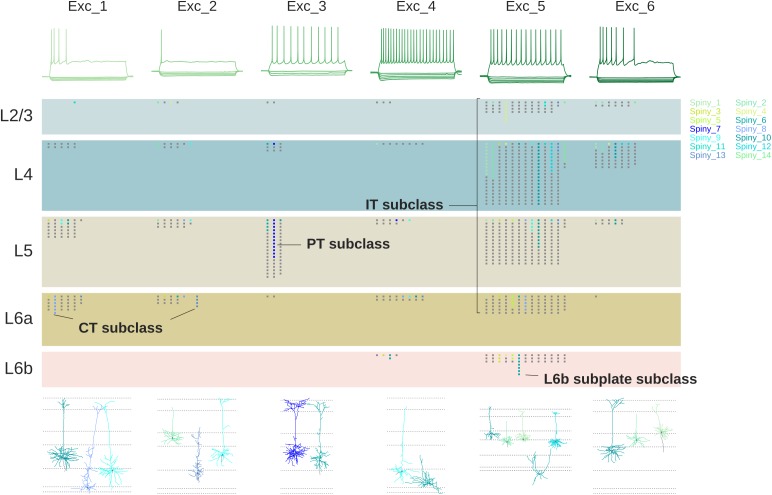
Relations between firing patterns, laminar localization, connectivity and morphology of pyramidal cells. Types of firing patterns at top are aligned with representative cell morphologies giving the patterns at bottom. Laminar localization is shown (each dot a recorded cell) in the middle separated into IT, PT and CT categories; colors indicate the reconstructed cells at bottom [Adapted from Gouwens et al., 2018].

Specific impulse firing patterns are necessary for giving insight into the neural basis of behavior. This information is obtained by a variety of recording methods, including extracellular single- and multi-electrodes in anesthetized or behaving animals, field potentials, and functional imaging, to name the most important. The Blue Brain Project has been leading the effort to identify these “morphoelectric” cortical neuron types. Firing properties are characterized by responses to injected current: the main response types are a burst (burst) of impulses; a steady non-adapting (non-adapt) impulse train; a rapidly adapting firing (rapidadapt); and fast spiking (fastspike) (see the different neuron types). Figure 7 shows an example of the relations between cell morphology, impulse firing pattern, cell layer, and connectivity for excitatory pyramidal neurons, from the study of Gouwens et al. (2018) at the Allen Brain Institute.

**FIGURE 8:**
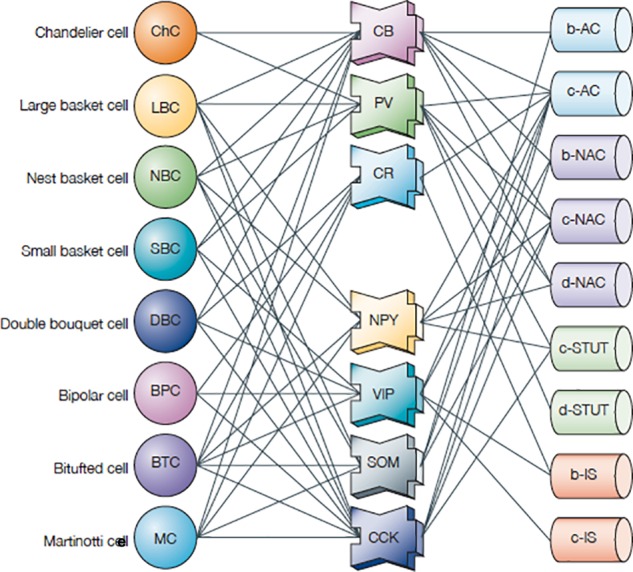
Interneurons (left column) in the neocortex: correlation of expression of calcium-binding proteins (CBPs: middle column: CB, calbindin; PV, parvalbumin; CR, calretinin NPY) and neuropeptides (NPY, neuropeptide Y; VIP, vasoactive intestinal peptide; SOM, somatostatin; CCK, cholecystokinin) with different morphological and electrophysiological classes (right column: AC, accommodating; b, burst; c, classic burst; d, delay burst; iS, irregular spiking; NAC, non-accommodating; STUT, stuttering) [Adapted from Markram et al. (2004)].

Figure 7 might suggest that a given morphological cell type is always associated with a given functional property, but as usual, biology isn’t that simple. As an example, Markram et al. (2004) identified eight morphological types of neocortical inhibitory interneurons. They found that all types expressed not just one, but several tags of calcium-binding proteins and neuropeptides. They went further and characterized the physiological properties in terms of 9 different impulse firing patterns. In about half the cases a given firing pattern was associated with more than one molecular tag and more than one morphological type. As can be seen in Figure 8, this made for a complex combinatorial pattern of associations between morphology, molecular identity, and firing pattern.

These complex patterns raise the question of whether neocortical interneurons contain a continuum of different combinations of properties or are divided into distinct classes. Markram et al. (2004, p. 804) note: “… only a few transcription factors, expressed in different combinations, might give rise to a finite number of distinct classes of interneuron. So, most interneurons probably lie in distinct electrical, morphological and molecular classes. The observed diversity is several orders of magnitude smaller than expected for a continuum of electrical types using more than 100 ion-channel genes, indicating powerful constraints on diversity. Understanding these constraints is also key to resolving the class-versus-continuum debate.”

Unique physiological properties are the most difficult to characterize as a neuron class; Markram et al. (2004) note:

“The proof that these responses represent distinct classes and that each class maps onto anatomically and molecularly distinct types of interneuron is still lacking … The fundamental question now is how microcircuits in different species, different brain regions of the same species, different layers and even different neurons in the same layer are driven to diversify to form countless variations of the microcircuit template – in particular, whether stimulus diversity is the ultimate driving force behind interneuron diversity.”

How does one name a neuron in which these issues have not yet been resolved? The parent-child approach may be useful. If a combination of gene expression, neuropeptides and functional properties is found to define a subset of neurons, it can be identified as such, either by its most dominant characteristic(s), by a single characteristic noted by an asterisk, or by a new symbol. For example, a basket cell in M1 L2/6, drawing on Figure 8, could be named:

**Table:** 

Region	Subreg	Layer	Conn	Trad. Name	Genes
neocortex	M1	L2/6	INT	basket	*Pvalb*
Peptides	Physiol	Trans
NPY	burst	GABA
SOM CCK		

It should be remembered that a known property carries with it the caution that it does not exclude the possibility that further research will reveal the expression of other properties, an important condition on any neuron name.

### Neurotransmitters

The functional properties of the neuron drive the output through synapses on other neurons. Chemical synapses are the primary means for communication between neurons. There are several types of neurotransmitter molecules: the main actors are glutamate (GLU), gamma-amino-butyric acid (GABA), acetylcholine (ACh), and dopamine (DA). As we have shown, these can easily be added to the name. The excitatory action of glutamate is of course not a property of the releasing neuron but of the receptors on its cell target; its inclusion in the name could be optional if it is relevant. Similarly, cortical interneurons are mostly GABAergic and inhibitory. More slowly acting modulators such as neuropeptides and neurohormones can also be added. Including these among the functional properties in the neuron name greatly facilitates identifying the neurotransmitter, neuropeptide and neurohormone families across the database, as has been done in NIF NeuroLex and NeuronDB.

The other main type of synapse is the gap junction (electrical synapse). It consists of a scaffold of proteins that connect cells to form pores allowing small molecules and electric current to flow between them. They are common in many cells; for example, between INTs in the neocortex. They can be indicated by “gap” added to the name, as in:

**Table:** 

Neocortex	M1	L2/6	INT	basket	burst
GABA	gap

## The Proposed Format Is Supported by Recent Gene Studies

A summary of the naming format for the neurons considered here is provided in Table 3. The advantages for investigations of single cells are evident in displaying both the relatively stable (anatomical) and functional properties, together with the most important genes for the identity of the cell type, without having to search a separate database. The advantages for listing cells from different regions in the same database are evident in the strict lineup of categories, facilitating comparison of a given cell type with other types within the same region and between different regions. The names shown for principal (pyramidal) cells and interneurons will apply to neurons in most cortical areas.

**Table 3:** Summary of formats for anatomical and functional properties of different neuron types covered in the text.

	Anatomical Properties				Functional Properties			
		
Region	Subreg	Layer	Conn	Trad. Name	Genes	Peptides	Physiol	Trans
Spinal cord	lumbar	VH	Skel	MN alpha	*Err3*		adapt	ACh
Spinal cord	lumbar	VH	INT	Renshaw			burst	GLY
Striatum	caudate	strio	direct	m. spiny	*Ppp1r1b*		non-adapt	GABA
Striatum	caudate	strio	INT	cholinergic	*CHAT*		burst	ACh
Piriform	anterior	supfl	af	pyramidal	*Cux1*		non-adapt	GLU
Piriform	anterior	supfl	INT	superfl int			burst	GABA
Hippocampus	dentate		MF	granule	*Math-2, Tox3*		non-adapt	GLU
Hippocampus	CA3		Sc	pyramidal	*Math-2, Coch*		non-adapt	GLU
Hippocampus	CA1		Sub	pyramidal	*Math-2, Wfs1*		non-adapt	GLU
Neocortex	M1	L2/6	IT	pyramidal	*Cux1, Satb2*		non-adapt	GLU
Neocortex	M1	L5b	PT	pyramidal	*Sox5-Fezf2*		non-adapt	GLU
Neocortex	M1	L6	CT	pyramidal	*Zfpm2*		non-adapt	GLU
Neocortex	M1	L2/3	INT	basket	*Pvalb*	SOM	burst	GABA
Neocortex	M1	L2/3	INT/P	basket	*Pvalb*	SOM	burst	GABA
Tasic et al., 2018								
Neocortex	M1	L5	IT	pyramidal	*Tnc*		non-adapt	GLU
Neocortex	M1	L2/6	INT	basket	*Reln, ltm2, Pvalb*		burst	GABA
Gillespie et al., 2018								
Neocortex	S1		INT	L basket	*Pvalb*	+VIP -SOM	fast spiking	GABA
Gouwens et al., 2018								
Neocortex	VISp	L4	IT	pyramidal	*Rorb*		adapt	GLU


*Single entries for genes and peptides are examples from larger populations which may be archived in separate databases. At the bottom for comparison are formats for current studies covered in the text of RNA-seq gene expression. The comparison shows the close similarity between the approaches, indicating that the format can accommodate new data regarding genes and peptides revealed by RNA-seq and other methods. Leaving blank the entries for genes and peptides when not yet available makes clear where new studies are needed, while also providing comparisons with what is known in other neuron types (see text). Abbreviations for Gouwens et al. (2018): VISp, VI S posterior; spiney 1 cluster, gene group; for other abbreviations, see previous nomenclature entries for these neurons.*

It may seem that the inventory for cortical pyramidal cells is unnecessarily repetitious because they all appear to have the same properties. However, they should be specific for each area because of the distinct connectivity of input fibers from different brain regions to each area, and the different output targets of the principal neurons of each area. Although differences in properties between neurons in different areas may not be apparent now, future research will test the extent to which they may be similar or different.

The format appears marred by the lack of data for genes and peptides for most of the cell types depicted, suggesting those categories could be deleted. The absences are in fact useful because they make specific the need for those data for those cells. The naming scheme can thus serve as a stimulus for investigations of those cell types, providing at the same time the context of what is known in the other cell types.

The table has the further advantage that it allows us to compare the approach used in this proposal with the approach used in the recent studies of gene expression that we have mentioned. As shown at the bottom of the table, the format for the expressed genes reported by Tasic et al. (2018) in fact fits precisely with the properties as far as they go into the proposed scheme. This applies to the properties for both pyramidal cells and interneurons. Similarly, the properties reported by Gillespie et al. (2018) and Gouwens et al. (2018) also fit very closely. These recent results show that the proposed naming format can also be applied to data-driven classifications of neuron types. While the name cannot capture the structure of the classifier, it still retains several of the salient features.

When we began our review these data were not available; the fact that the new data fit so well indicates that the format is likely to prove effective for future work. These new reports enter the era of high throughput RNA-seq, generating up to hundreds of genes, which obviously do not fit into the name. The focus should be on the expression of those genes most essential to the identity of that neuron type. An example would be the Sox5 – Fezf2 transcription factor regulatory network expressed in subcerebral (PT) pyramidal cells (see Table 3). Otherwise there needs to be a link to a database of these high capacity studies. Databases are being constructed for this purpose.

## Multimodal Searches for Families of Properties

We have focused on the unique set of properties shared by all members of a given neuron morphological type. However, neurons can also be classified by their shared functional properties independent of their anatomical shape.

Exploring this possibility is analogous to identifying families in sequence databases using the powerful tool in bioinformatics called Basic Local Alignment Search Tool (BLAST). This enables a search of a gene or protein database for any arbitrary sequence of nucleotides or amino acids, to identify families with shared properties that otherwise are unknown. This constitutes a single modality BLAST search.

A novel multimodal tool has been created by SenseLab to enable an analogous search of different neuron properties, by membrane currents, neurotransmitters, and neurotransmitter receptors that are contained in CellPropDB and NeuronDB. In analogy with BLAST, this can be termed a Multimodal Alignment Search Tool (MAST). The power of a MAST search is that one can take an arbitrary combination of currents, receptors and/or transmitters found in a cell of interest and search CellPropDB or NeuronDB for the family of neurons containing the same properties. An obvious example is the family of all the neurons that express glutamate or gaba. This is also possible in the NIF Neurolex. Even more precisely, in NeuronDB one can search for the families of currents, receptors and/or transmitters found in a specific axon, soma or dendritic compartment. Such across-neuron families imply that these morphologically distinct neurons and neuron compartments carry out similar processing operations, as has been shown in a previous study (Migliore and Shepherd, 2002). Understanding these common functional motifs across morphologically different neurons and neuron compartments will become increasingly important with increasing research on functional properties at the cellular and subcellular level. It is an additional reason to have these properties be explicit in the neuron name alongside the traditional morphological features. Effective use of this tool depends on population of the searched databases, which is in progress.

## State Dependence of Neuronal Properties

The functional properties of a brain region are state dependent: they vary depending on the behavioral state of the animal. When the focus is on anatomical features as the basis of nomenclature this fact is usually overlooked. However, when the nomenclature reflects functional properties they must be taken into consideration. A typical example; zebrafish fast motor neurons may secrete glutamate plus ACh during forced exercise (Bertuzzi et al., 2018). Thus, the functional properties of a pyramidal cell are different whether the animal is active or resting; awake or sleeping; hungry or sated; sexually active or not; an alpha or beta male; estrous or menopausal; normal or addicted; responding to injury; and so on. This also applies to gene expression; the expression of individual genes in individual cells also varies with many of these behavioral or cognitive states. A nomenclature must regard these added complexities not as problems but as opportunities to reflect the nervous system as it really is.

## Development

We have seen that during evolution there was continuity of the three main types of cortical pyramidal neurons when characterized in terms of connectivity: intratelencephalic, pyramidal tract and corticothalamic. It remains to ask how these types emerge during early development and are maintained into adulthood, a field of increasingly intense activity. Cortical neurogenesis and cell type specification and maintenance depend on networks of transcription factors, regulatory elements, synaptic interactions and modulatory signals. In the summary diagram of Figure 9 from Shibata et al. (2015), different types of pyramidal neurons are sequentially generated by the same lineage of progenitor cells in the ventricular and subventricular zones (VZ and SVZ, respectively) and migrate into the emerging cortical plate in “inside first, out last” manner. Birthdating and lineage studies have shown that the earliest ascending cells form the large deep layer pyramidal cells shown on the right, whose axons constitute the subcerebral projections to the pyramidal tract (PT) and the thalamus (CT). In contrast, later ascending cells differentiate into pyramidal cells which distribute themselves mainly in the upper layers but also throughout; these become the intracerebral (intratelencephalic: IT) pyramidal cells. The proposed cortical pyramidal cell nomenclature is thus consistent through early development (this figure), adult connectivity (Figure 6), and evolution (IT cells in Figure 3) (cf also Table 3). Our review of cortical names has thus taken us to the earliest mechanisms for when and how the different neuron types arise.

**FIGURE 9:**
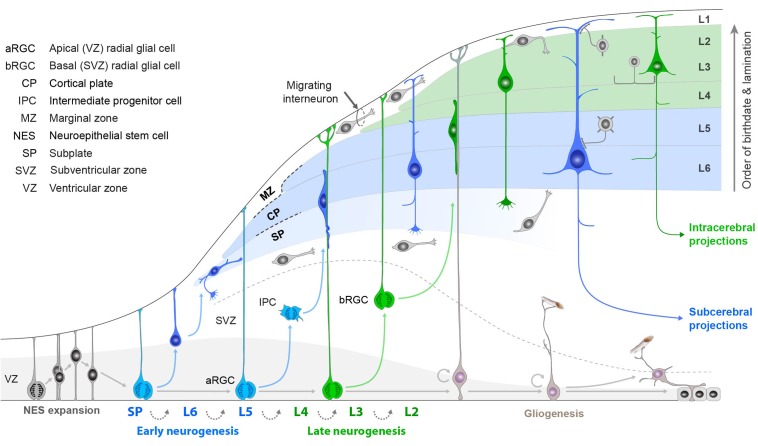
Summary of steps in neurogenesis and differentiation of the main types of neocortical pyramidal cells based on intracerebral (intratelencephalic IT) and subcerebral (pyramidal tract PT, corticothalamic CT) connectivity, as described in the text. Note the consistency of these types with the adult pyramidal cell types in Figure 3, 4, and 6. [Adapted from Shibata et al. (2015); see also Rakic (2009)].

In summary, complex gene networks, varying with activity and developmental stage, arise early in development to construct a neuron’s identity as reflected in its name.

## Discussion

In conclusion, we summarize the advantages of moving toward a systematic format for neuron names in which traditional names based on structure are extended to include genes and functional properties.

First, the format builds directly on classical terminology and on current initiatives in terminology, knowledge bases, and databases as in NeuronDB, NeuroMorpho.Org, Hippocampome.org, NIF, and BAMS. Most of the traditional names are still identifiers for each main neuron type.

Second, it anchors each neuron type to the region in which its cell body is localized. There is no ambiguity, for example, about whether a “granule cell” is in the cerebellum, olfactory bulb, neocortex, dorsal cochlear nucleus, or dentate gyrus. This enhances archival listing in a database because it enables easy alphabetical order by “parent-child relation,” grouping all cells in a given region instead of distributing them throughout the database due to spelling of modifying terms. This approach is already established in NeuronDB and the NIF. The format also enhances research on an individual neuron type by making all properties visible so that new properties can readily define new neuron subtypes.

Third, the approach differentiates long-axon “principal” cells from short-axon “interneuron” cells, a distinction going back to Golgi and in wide use today, crucial to understanding the distinctive functions that these two types have in information processing. Both types carry out local processing; the long axons also transmit specific information between regions while the short axons function mainly as modifiers within regions.

Fourth, in cortical areas, the pyramidal cell functions as the core of a basic cortical module in cortical evolution, with recurrent and lateral excitation and inhibition, together with modulatory interneurons, laid down during development and present across the vertebrate series.

Fifth, the neocortical pyramidal cells, as principal neurons carrying cortical output, can be characterized in two ways within the neuron name. One is by the location of their cell body in one of the different cortical layers, recognizing that a layer may contain different cell types. The other is by the connectivity of their axons, of which there are three main types: intratelencephalic (IT), connecting to ipsilateral and/or basal ganglia and neocortex; pyramidal tract (PT), connecting to the pyramidal tract and the neuraxis; and corticothalamic (CT), connecting to the thalamus. The relevance of this dual characteristic is seen in its current use in identifying the expression of genes in cortical pyramidal cells.

Sixth, genes and functional properties fit naturally into the expanded name for a neuron. These should include labels for the neurotransmitter released by the neuron; neuropeptide neuromodulators that are markers for the neuron type; significant genes expressed by that type; and characteristic impulse firing patterns associated with that type. Including these in the name for the neuron makes its multiple properties immediately and unambiguously recognizable. As a result, the multimodal properties that define a specific neuron type are present in the name itself, making its anatomical, genetic and functional identity immediately obvious.

Seventh, a succinct terminology format is suggested in which the basic properties are indicated, beginning with the general and relatively more stable defining anatomical characteristics of region, subregion, and connectivity, and adding more detailed functional properties including neuropeptides, gene markers, physiological firing patterns, and neurotransmitters.

Eighth, with functional properties carried transparently in the name across different anatomical neuron types, there will be an enhanced ability to identify functional motifs that carry out similar processing steps despite different morphologies and connectivities. These functional families should go far beyond the present recognition of glutamatergic excitatory and GABAergic inhibitory cells, for example, and reveal similar or contrasting basic processing steps carried out by different neurons and their subcellular compartments at all stages of development and aging.

Ninth, in a rapidly evolving field such as neuroscience, one of the challenges is having names for neurons remain stable despite new research constantly revealing new functional properties. One way this is accomplished is by having the category of relatively stable, anatomically based neuron types provide the basic family identity, so that when new research expands the number of functional subtypes they are all children of the same parent, i.e., they form an extended family.

Tenth, the neocortex presents a special problem in that, because of the multiple cortical areas, multiple layers, and multiple output connections, the number of distinct neuron types could exceed 1,000 for just the adult mouse, and many times that for the human. This uniquely large number reflects one of the essential features of the neocortex: the multiple neuron types within a cortical region are potentially able to process information in many different ways, inborn or learned, in generating the rich behavioral repertoire of the animal. Special databases will be required to give adequate recognition to the especially complex neuron populations in the neocortex, and for other complex regions such as the retina, similarly to recent and ongoing progress for the hippocampus.

Finally, the new era of high-throughput RNA-seq and its related methods is yielding massive data sets of gene expression that go far beyond previous characterizations of neuron types. Separate databases will obviously be needed for these gene data. In addition, variation of gene expression within a type is challenging the very concept of a neuron type, as we have documented and is summarized well by Cembrowski and Menon (2018):

“Next-generation RNA sequencing (RNA-seq) is becoming increasingly popular in the deconstruction of this complexity into distinct classes of ‘cell types’ … the technology has also begun to illustrate that continuous variation can be found within narrowly defined cell types. Here we summarize the evidence for graded transcriptomic heterogeneity being present, widespread, and functionally relevant in the nervous system. We explain how these graded differences can map onto higher-order organizational features and how they may reframe existing interpretations of higher-order heterogeneity. Ultimately, a multimodal approach incorporating continuously variable cell types will facilitate an accurate reductionist interpretation of the nervous system.”

## Author Contributions

GS developed the idea in discussions with LM, MH, MM, RM, NC, AN, MS-Z, and GA. GS wrote the first draft of the manuscript. GS and GA finalized the manuscript after approval by the co-authors.

## Conflict of Interest Statement

The authors declare that the research was conducted in the absence of any commercial or financial relationships that could be construed as a potential conflict of interest.

## Footnotes

1 https://senselab.med.yale.edu/

2 https://senselab.med.yale.edu/NeuronDB/

3 http://mouse.brain-map.org/static/atlas
